# Identification of Circulating Biomarker Candidates for Hepatocellular Carcinoma (HCC): An Integrated Prioritization Approach

**DOI:** 10.1371/journal.pone.0138913

**Published:** 2015-09-28

**Authors:** Faryal Mehwish Awan, Anam Naz, Ayesha Obaid, Amjad Ali, Jamil Ahmad, Sadia Anjum, Hussnain Ahmed Janjua

**Affiliations:** 1 Atta-ur-Rahman School of Applied Biosciences (ASAB), National University of Sciences and Technology (NUST), H-12 Islamabad, Pakistan; 2 Research Center for Modeling and Simulation (RCMS), National University of Sciences and Technology (NUST), H-12 Islamabad, Pakistan; University of Salerno, Faculty of Medicine and Surgery, ITALY

## Abstract

Hepatocellular carcinoma (HCC) is the world’s third most widespread cancer. Currently available circulating biomarkers for this silently progressing malignancy are not sufficiently specific and sensitive to meet all clinical needs. There is an imminent and pressing need for the identification of novel circulating biomarkers to increase disease-free survival rate. In order to facilitate the selection of the most promising circulating protein biomarkers, we attempted to define an objective method likely to have a significant impact on the analysis of vast data generated from cutting-edge technologies. Current study exploits data available in seven publicly accessible gene and protein databases, unveiling 731 liver-specific proteins through initial enrichment analysis. Verification of expression profiles followed by integration of proteomic datasets, enriched for the cancer secretome, filtered out 20 proteins including 6 previously characterized circulating HCC biomarkers. Finally, interactome analysis of these proteins with midkine (MDK), dickkopf-1 (DKK-1), current standard HCC biomarker alpha-fetoprotein (AFP), its interacting partners in conjunction with HCC-specific circulating and liver deregulated miRNAs target filtration highlighted seven novel statistically significant putative biomarkers including complement component 8, alpha (C8A), mannose binding lectin (MBL2), antithrombin III (SERPINC1), 11β-hydroxysteroid dehydrogenase type 1 (HSD11B1), alcohol dehydrogenase 6 (ADH6), beta-ureidopropionase (UPB1) and cytochrome P450, family 2, subfamily A, polypeptide 6 (CYP2A6). Our proposed methodology provides a swift assortment process for biomarker prioritization that eventually reduces the economic burden of experimental evaluation. Further dedicated validation studies of potential putative biomarkers on HCC patient blood samples are warranted. We hope that the use of such integrative secretome, interactome and miRNAs target filtration approach will accelerate the selection of high-priority biomarkers for other diseases as well, that are more amenable to downstream clinical validation experiments.

## Introduction

Hepatocellular carcinoma (HCC), one of the most aggressive and devastating cancer, with an annual incidence of 0.6 million new cases, is the third leading cause of cancer related mortality worldwide [1, 2]. Its incidence remains highest in the developing world and is steadily increasing across the developed world. Early diagnosis and metastasis monitoring of HCC still remains a challenging task and is therefore highly important [3]. In most of the cases, HCC patients die quickly because of the late diagnosis and rapid tumor progression. Hepatic resection and liver transplantation are the only potential curative treatments for HCC patients [3]. Even after curative resection, HCC recurrence occurs in 60–100% of the cases thus limiting the long-term survival of HCC patients. Biomarkers in blood or in other body fluids for screening, staging, prediction of recurrence, prognosis and monitoring of response to a therapy would be an important contribution to the management of patients with HCC.

Biomarkers, as quantifiable traits can evaluate normal biological as well as pathological processes [4]. Detection of tissue-specific circulating biomarkers to find tumor at an early stage and to enable minimally invasive monitoring of patient health states have gained immense scientific and clinical value. Various tumor-related genes, proteins, enzymes and microRNAs (miRNAs) synthesized by the cancer tissues are secreted into the body fluids such as blood or urine. They can be measured by non-invasive assays [3] and thus are considered to be rich sources of potential biomarkers [5]. Currently, the level of serum alpha-fetoprotein (AFP) is being used as a standard biomarker for the diagnosis of HCC with ultrasonography every 6 to 12 months [1]. The diagnostic performance of AFP is heavily constrained due to its low specificity and sensitivity which significantly reduces its reliability in clinical settings and is therefore not recommended in the current American Association for the Study of Liver Diseases (AASLD) guidelines [6]. Diagnostic accuracy of AFP is usually impaired due to its high cell turnover which is often seen in patients with inflammatory active, HCV-associated liver cirrhosis. Therefore, integrated multidisciplinary research focusing on highly specific and sensitive circulating biomarkers to detect HCC at an early stage can have a profound and significant effect on increasing patient survival rate [7, 8].

Bioinformatics as a new emerging technology has the capacity to revolutionize biomarker discovery by linking scientific data with clinical information. Meta-analysis efforts are scaled up via searchable databases that motivate biologists and clinicians to aggregate data across various studies. Since limited size is the major hurdle in studies involving human subjects, meta-analysis methods that seek to improve the detection of reliable biomarkers through aggregation of various datasets have received considerable attention. Furthermore, the quantitative proteomics usually generates a huge amount of data that needs to be further analyzed in order to identify marker candidates. Here, we propose a multi-step prioritization process for the identification of potential circulating HCC biomarkers via a comprehensive *in-silico* secretome and interactome analysis along with HCC-specific circulating and liver deregulated miRNAs target filtration for experimental evaluation. Major emphasis in the current study was on secretome analysis, as most of the tumor biomarkers are likely to be found in the secretome (body fluids) [9, 10]. We designed a biomarker prioritization approach which begins with data extraction from seven publicly available gene and protein databases for the selection of liver-specific proteins. These databases describe the expression of thousands of genes and proteins in multiple tissues and allow investigators to select candidate markers with higher tissue specificity based on their relative expression pattern [11]. Following the selection of liver-specific proteins, secretome analysis was performed to sort out secreted or shed proteins. The *in-silico* pipeline for the prediction of secreted proteins provides a rapid screen to identify biomarkers that are found extracellularly and likely to be detectable by non-invasive assays. The secreted proteins identified in current study were further filtered by integrating proteomic datasets enriched with liver cancer secretome [12]. Identified candidate biomarkers were further sorted via interactome analysis with current standard HCC biomarker AFP and its interacting partners to assess possible involvement in HCC pathogenesis. Interactome anlaysis with dickkopf-1 (DKK1) and midkine (MDK) was also performed as these two serum biomarkers are considered more specific and sensitive than currently used biomarker AFP [13, 14]. A final selection criterion in our prioritization strategy was HCC-specific circulating and liver deregulated miRNAs target filtration. miRNAs control the expression of several genes which can be of high importance in biomarker validation. The candidate proteins were further analyzed on the basis of their encoding genes and observed whether they are validated targets of HCC-specific circulating miRNAs or not. We believe that proteins identified through our proposed approach are highly specific and sensitive which can serve as potential circulating biomarkers for detection and prognosis of HCC. Moreover, we propose a generalizable approach that could speed-up biomarker discovery and can be applied in bulk to public datasets to achieve improved results in various other cancers and diseases as well. We believe that by using existing and emerging computational data mining approaches for rigorously and systematically evaluating different types of genomic and proteomic information will increase the probability of finding out highly potential biomarker candidates.

## Materials and Methods

### Microarray, immunohistochemistry (IHC) and expressed sequence tags (ESTs) data processing

Seven gene and protein databases (S1 Table) based on the data extracted from microarray, IHC and ESTs experiments were mined to identify proteins highly specific to and strongly expressed in liver. The C-It database [15] was used for proteins enriched in liver. The C-it database is based on the Database-dependent Gene Selection and Analysis (DGSA) algorithm. This algorithm identifies tissue-enriched genes by using EST profiles in all available tissues of organisms. C-It combines microarray and SAGE data to give users integrated access to comprehensive transcriptional profiles. Furthermore, C-It is linked with custom version of exon array analyzer to allow tissue-enriched alternative splicing analysis. Only proteins with corresponding SymAtlas z-score of ≥ 1.96 that reveals 95% confidence level of enrichment were included. Proteins without a SymAtlas z-score were ignored. The TiGER database [16] which provides and summarizes large scale data sets for tissue-specific gene expression and regulation in a variety of human tissues was used for proteins preferentially expressed in liver based on ESTs by searching liver tissue using ‘Tissue View’. The TiGER database contains three types of data including cis-regulatory module detections, tissue-specific gene expression profiles and combinatorial gene regulations. The UniGene database was searched for tissue restricted genes using the following search criteria: [liver] [restricted] + “Homo sapiens”. UniGene computationally identifies transcripts from the same locus; analyzes expression by tissue, age, and health status; and reports related proteins (protEST) and clone resources. The BioGPS database [17], a gene annotation portal based on a loose federation of existing genetic and genomic resources was also used. The BioGPS database plugin ‘Gene expression/activity chart’ using the default human data set ‘GeneAtlas U133A, gcrma’ was searched with a protein whose gene expression profile using the BioGPS plugin showed it to be specific to and strongly expressed in liver. For each protein searched, a correlation cutoff of 0.9 was used. BioGPS allows users to easily explore the landscape of gene annotation resources for one or more genes of interest. BioGPS is based on a simple, unstructured plugin interface that allows for simple community extensibility to harness the principle of community intelligence toward the goal of efficiently organizing and querying online gene annotation resources. TiSGeD database [18] was searched for proteins enriched in liver with SPM value of 0.9. SPM is a statistical parameter which serves as a sensitive indicator in quantitative estimation of gene spatial expression patterns. Liver tissue was searched in the VeryGene database [19] using ‘Tissue View’ for liver-selective proteins. The VeryGene database is curated, web-accessible centralized database for the annotation of tissue-specific/enriched genes. This database being configured into tissue view and gene view, retrieve information on tissue/subcellular localization, drug-disease relation and functional annotation. The HPA [20–22] was searched for proteins strongly expressed in normal liver tissue with annotated expression. Proteins identified in only one database were eliminated whereas those identified in two or more databases were selected as they could represent more promising candidates at this stage.

### Pipeline for the identification of secreted or shed proteins

Computational tools have been designed to assess proteins that follow either classical or non-classical secretory pathways. Many proteins are secreted by a classical secretory mechanism, i.e., with signal peptide (an N-terminal peptide, typically 15–30 amino acids long), which is cleaved off during translocation of the protein across the membrane, and can be predicted using the amino acid sequence of the protein. Prediction of secretory proteins was carried out using a pipeline of five tools; SignalP 4.1 [23], SecretomeP 2.0 [24], ExoCarta [25], TargetP 1.1 [26] and TMHMM v. 2.0 [27]. In the first step, the amino acid sequences of proteins were retrieved from the UniProtKB database [28] in FASTA format. Classical secretory proteins with a signal peptide were predicted by SignalP 4.1 server and were selected on the basis of their D-value above 0.45. SignalP server is considered to be most accurate method for the prediction of cotranlsationally translocated proteins (proteins entering the classical secretory pathway via the endoplasmic reticulum) [29]. Non-classical secretory proteins without a signal peptide were predicted by SecretomeP 2.0server and were selected by their neural network (NN) score ≥ 0.5. The method is also capable of predicting signal peptide containing secretory proteins in which only the mature part of the protein has been annotated or cases in which the signal peptide remains uncleaved. The identified liver-specific proteins were also searched against ExoCarta database [25] to determine whether they were present in exosome fractions or not. The combined set of SignalP, SecretomeP and Exocarta predicted proteins were passed to TargetP 1.1 for the exclusion of mitochondrial proteins. TMHMM v. 2.0 was used for the prediction of transmembrane proteins with default options. TMHMM server is currently considered to be the best performing transmembrane prediction program [30]. Predicted secretory proteins with no transmembrane helices were selected for further filtration.

### Verification of expression profiles in liver and blood

Expression profile verification of the secreted or shed proteins in liver and blood was done via pipeline of three databases; BioGPS, HPA and plasma proteome database. For liver tissue in BioGPS database, proteins with gene expression profiles showing similar values of expression or strong expression in other tissues along with liver tissue were eliminated (strong expression is defined as ≥ 10 times the median expression value in all tissues). In BioGPS, the color of the bars in the ‘Gene expression/activity chart’ reflects a grouping of similar samples, based on global hierarchical clustering. In order to systematically investigate the protein expression in cancerous versus normal tissues and cell type, the HPA is a most comprehensive resource because it includes millions of high-resolution IHC images with expert-curated annotations. HPA was used for qualitative comparison of IHC staining of liver cancer tissue with normal liver. HPA is an antibody-based database. Tissue microarray and IHC staining techniques are applied in HPA and it has comprehensively accumulated millions of high-resolution images with expert-curated annotations. IHC staining is regarded as an effective technique in proteomic research. On the basis of these images, especially those using IHC staining, the HPA has been effectively used in a number of studies for cancer marker discovery. Plasma proteome database [31, 32] was used next for further filtration of candidate biomarkers. The database was developed as a part of Human Proteome Organization and is one of the largest resources on proteins reported in plasma and serum.

### Integration of liver secretome proteome datasets

Secretome proteomes are rich source of circulating biomarkers therefore in current study we integrated various secretome studies conducted on HCC liver tissues, serum samples of HCC patients and HCC cell lines in order to filter only those proteins which have been detected in their secretomes. In biological research, mammalian cell lines are chosen to examine protein function and cell response to perturbations and these cell lines are indispensable for many of the biological insights. In the majority of the cases, these cell lines were extracted from tumors of different origins, and were then adapted to growth in vitro and therefore serve as proxies not only of the original tumors or tissues but also for fundamental biological processes [33]. The proteomes of cell lines can highlight the biological processes and their variations across the cells. In addition, the secretome signature of a cancer cell line can be considered a potential tool to investigate tumor aggressiveness and a preclinical exploratory study required to optimize the search of cancer biomarkers. Dealing with a cell-specific secretome limits the contamination by the major components of the human serum and reduces the range of dynamic concentrations among the secreted proteins, thus favouring under-represented tissue-specific species. Such a characterization allowed corroborating the potential of a cell culture-based model in order to describe the cell-specific invasive properties and to provide a list of putative cancer biomarkers [34]. The characterization of various cell lines showed that they are, in fact, an excellent model for the study of the biological mechanisms involved in cancer. The use of cancer cell lines allowed an increment of the information about the deregulated genes and signaling pathways in this disease. These cell lines are appropriate in vitro models in cancer research and are crucial for the investigation of potential molecular markers and for the screening and characterization of cancer therapeutics [35]. The data of proteomes from the conditioned media of 23 cancer cell lines (from 11 cancer types), characterized using one-dimensional SDS-PAGE and nano-liquid chromatography tandem mass spectrometry on a LTQ-Orbitrap mass spectrometer [36]; secretome of 12 individual paired samples of liver cancer and adjacent normal tissues analyzed by tandem mass spectrometery [37]; secretome of cholangiocarcinoma (HuCCA-1) and hepatocellular carcinoma (HCC-S102, HepG2, SK-Hep-1, and Alexander) cell lines analyzed by SDS-PAGE combined with LC/MS/MS [38]; secretome of hepatoma HepG2 cells characterized using two-dimensional liquid chromatography coupled with tandem mass spectrometry (2D LC-MS/MS) analysis [39]; secretomes of 21 cancer cell lines derived from 12 cancer types analyzed by SDS-PAGE combined with MALDI-TOF MS [40]; secretome of primary human hepatocytes (PHH), HepG2 and Hep3B cells analyzed by 2D-PAGE and shotgun proteomics [41]; serum analysis from patients with varying degree of hepatic scarring induced by infection with the hepatitis C virus based on 2-dimensional gel electrophoresis [42], proteome of HCC patients serum samples characterized using chromatography and tandem MS combined with iTRAQ [43]; serum proteome of 12 HCV related HCC patients characterized using reverse phase HPLC and SDS-PAGE [44] were integrated.

### Interactome analysis and miRNA target filtration

Protein-protein interactions (PPIs), being critical regulatory events are useful for associating proteins with diseases, fathoming signaling cascades and predicting protein functions. In order to determine whether the identified proteins interact with each other as well as with MDK, DKK1, current standard HCC biomarker AFP and its interacting partners, network based tools: STRING [45], FpClass [46] and GeneMANIA [47] were used. Functional links between proteins can often be inferred from genomic associations between the genes that encode them: groups of genes that are required for the same function tend to show similar species coverage are often located in close proximity on the genome and tend to be involved in gene-fusion events. The STRING database is a precomputed global resource for the exploration and analysis of these associations. FpClass database predicts high confidence experimentally predicted PPIs by identifying sets of features (e.g domains, posttranslational modifications, compatible domains that mediate interactions etc). GeneMANIA is a large collection of networks that are functionally associated (protein and genetic interactions both physical and predicted, pathways, protein domain similarity, coexpression and colocalization). Genes encoding candidate proteins identified were used for GO (Gene Ontology) analysis and KEGG (Kyoto Encyclopedia of Genes and Genomes) analysis via string database. GO analysis was applied to analyze the primary function of the differentially expressed genes according to GO, which is the key functional classification of the National Centre for Biotechnology Information (NCBI). Similarly, pathway analysis was used to determine the most significant pathway of the differentially expressed genes according to KEGG.

**Fig 1:**
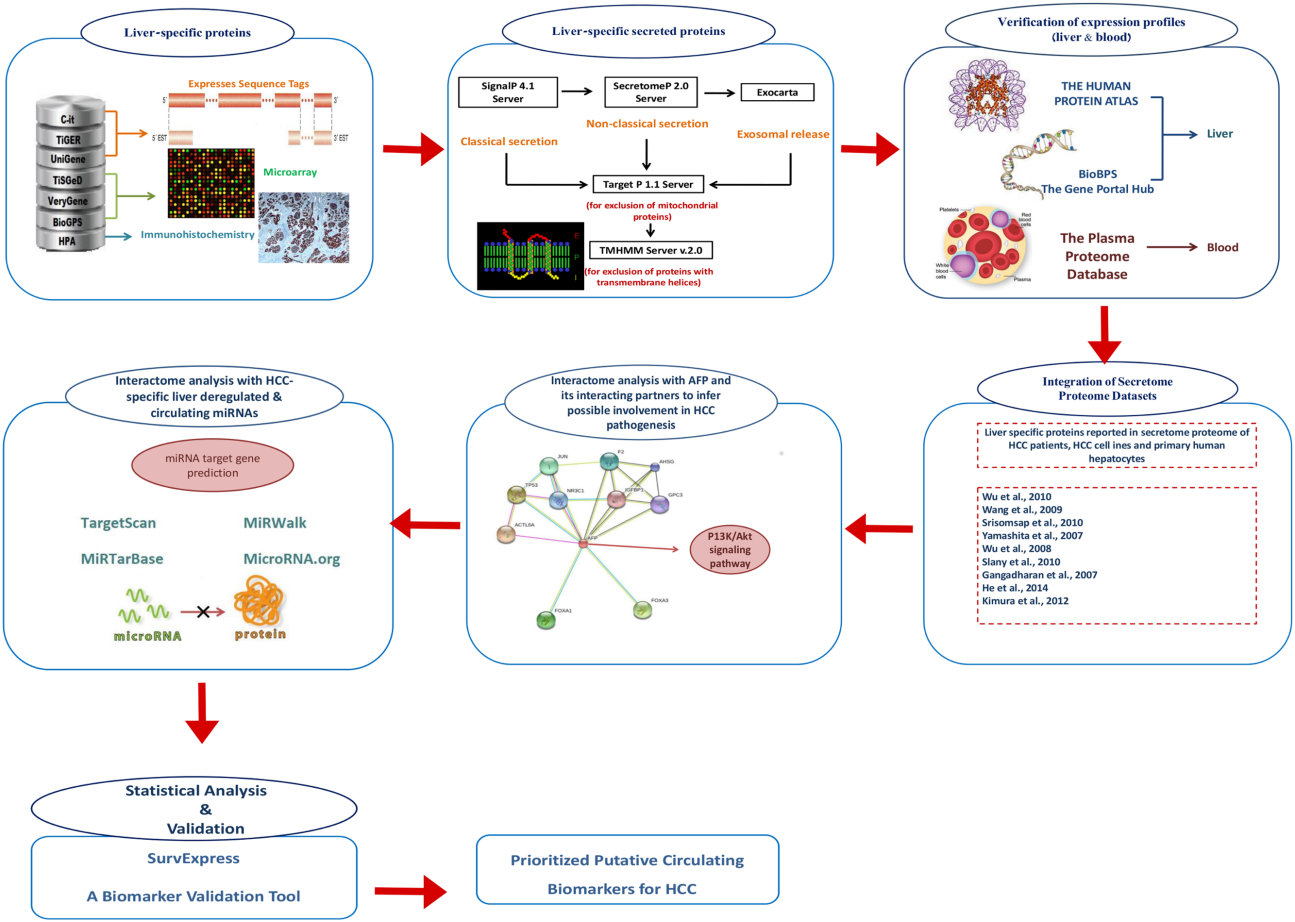
Schematic outline of multi-step HCC circulating biomarkers prioritization process. Liver-specific proteins extracted from various databases were screened using SignalP 4.1, SecretomeP 2.0, ExoCarta, TargetP 1.1 and TMHMM v. 2.0 servers to assess their secretory nature. Liver-specific secreted proteins once verified for their expression in liver (HPA and BioGPS) and blood (Plasma Proteome Database) were further prioritized depending upon their presence in secretome proteome of HCC patients, HCC cell lines and primary human hepatocytes. To infer possible involvement of prioritized proteins in HCC pathogenesis, their interactome analysis was done with AFP (as a standard biomarker for the diagnosis of HCC). Interacting proteins were then analysed for their interaction with HCC specific liver deregulated and circulating miRNA. Results were then statistically verified using SurvExpress validation tool to finally prioritize putative circulating biomarkers for HCC.

In order to determine whether the candidate proteins are encoded by the target genes of HCC-specific deregulated miRNAs, miRWalk [48], miRTarBase [49], TargetScan and microRNA.org [50] databases were used. Various studies have reported that differential expression of miRNAs can affect the expression of their target genes, leading to changes in the levels of the proteins they encode. Study conducted by Wang et al. on sepsis patients showed that genes encoding proteins ACVR2A, FOXO1, IHH, STK4 and DUSP3 were found to be the targets of the six miRNAs (miRNA miR-223, miR-122, miR-15a, miR-483-5p, miR-16 and miR-193b*). The expression profiles of these proteins were negatively correlated with above mentioned six serum miRNA levels (Wang et al., 2014a). Cytoscape network analysis platform [51] was used for the construction of interactome network. Cytoscape software provides basic functionality for visualizing, modeling and analyzing molecular and genetic interaction networks as well as integrating the network with expression profiles, phenotypes, and other molecular states; and to link the network to databases of functional annotations. A computational framework of whole prioritization strategy is given in Fig 1 and list of various databases/tools used in current study are given in S1 Table.

### Statistical analysis

Validation of putative biomarkers based on statistical methodology must find out associations, established by authenticating its correlation with clinical outcome. Validated biomarkers can improve clinical diagnosis, serve as useful prognostic and predictive factors of clinical outcome as well as lead to targeted therapies. Assessing the performance of proposed candidate biomarkers in different populations or evaluating competing biomarkers are challenging tasks. For scrutinization and validation of biomarkers, tools as ITTACA [52], RecurrenceOnline [53], GOBO [54], PrognoScan [55] and bc-GeneExMiner [56] have been proposed. However, these tools have serious restrictions and limitations. SurvExpress [57], Compared with other tools, is the largest and the most versatile free tool to perform validation of multiple biomarkers in human cancers, collecting more than 20,000 samples and 130 datasets with censored clinical information covering tumors over 20 tissues. Therefore, SurvExpress validation tool was used to identify statistical significance of proposed candidate circulating biomarkers for overall survival, HCC-free survival, relapse-free survival as well as the ability to discriminate from cirrhotic patients. In order to analyze the performance of our proposed biomarkers in relation to HCC-free survival rate as well as relapse-free survival rate, ROC curves (using Kaplan—Meier (KM) and Nearest Neighbor Estimation (NNE) methods) by analyzing the area under the curve (AUC) were calculated. In a ROC curve, each point represents a sensitivity/specificity pair corresponding to a particular decision threshold by plotting the true positive rate (Sensitivity) in function of the false positive rate (100-Specificity).

In order to assess whether proposed biomarkers can discriminate HCC from cirrhosis we performed ROC analysis to determine correlation of the proposed biomarkers with cirrhosis. Patients’ data of HCC (with hepatitis) and cirrhosis (with hepatitis) was taken from survExpress dataset (162 samples) (Hoshida Golub Liver GSE10143) which includes data from patients with tumor (HCC) and non-tumor (with cirrhosis).

SurvExpress validation tool accomplishes multivariate survival analysis and risk assessment of cancer datasets via Kaplan—Meier Plot and log-rank test. Kaplan-Meier Plot is a graphical representation of the survival probability (vertical axis) versus time (horizontal axis) estimated with data using

$$S\left(t_{i}\right)=S\left(t_{i}-1\right)*\frac{\left(n_{i}-d_{i}\right)}{n_{i}}$$

Where,

*S(t*_*0*_ *)* = 1,

*t*_*i*_ is i-th observed time.

*d*_*i*_ is the number of events at time *t*_*i*_ (deaths) and

*n*_*i*_ is the number of individuals not having the event (alive) just before *t*_*i*_ (assuming ordered times *t*_*i*_).

As a result, a staggered curve is generated, which represents the fraction of deaths in every stage known as instantaneous hazard. Whereas, the Log-rank test has been proposed to statistically evaluate the equality of survival curves.

Utilizing SurvExpress tool, overall survival, HCC-free survival and relapse-free survival (RFS) functions were compared using Kaplan—Meier estimates and statistical significance was determined using the log-rank test. Kaplan Meier plot includes the Concordance Index (CI) and the p value testing for equality of survival curves using a log rank test, and the correlation coefficient estimated from deviance residuals. The CI estimates the probability that subjects with higher risk prediction will experience the event after subjects of lower risk. CI is a generalization of the AUROC used in classification problems. The CI is expressed as:

CI=1|Ω| ∑ i,j∈Ω {1 if ri>rj 0 otherwise

Where

*ri* = the risk predictors given by the corresponding prognostic index for subjects *i*.

*rj* = the risk predictors given by the corresponding prognostic index for subjects *j*.

*Ω* = all subjects pairs (*i*, *j*) where *ti < tj* and subject i is not censored.

As in AUROC, CI values close to 0.5 are putatively ‘random’ whereas higher values are associated with better prediction.

## Results

### Liver-specific proteins

**Fig 2:**
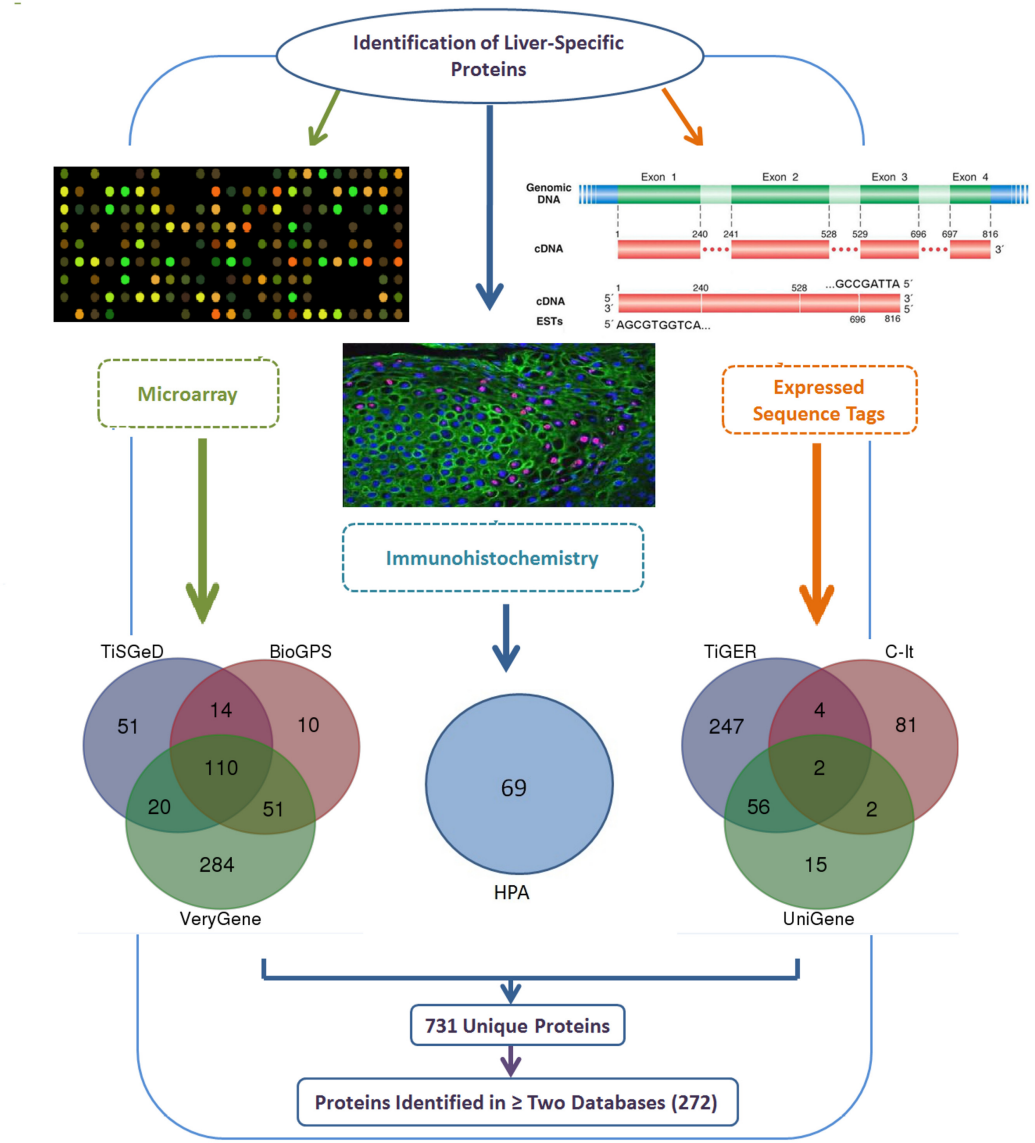
Identification of liver-specific secreted proteins. Liver-specific secreted proteins identified using seven publicly available gene and protein databases. Databases based on microarray data (TiSGeD, BioGPS and VeryGene) unveiled 845; ESTs data (TiGER, UniGene and C-It) revealed 473 and HPA database based on immunohistochemistry data revealed 69 liver-specific proteins. A total of 272 proteins were identified in two or more than two databases and thus selected for further analysis.

Seven gene and protein databases (S1 Table) used in the current study identified 731 proteins that were highly specific and strongly expressed in liver (Fig 2). The C-It database identified 89 liver-enriched proteins, the TiGER database identified 309 proteins preferentially expressed in liver and the UniGene database identified 75 liver-restricted proteins. The BioGPS database identified 185 proteins similarly expressed as protein with known liver specificity, the VeryGene database identified 465 liver-specific proteins and the TiSGeD database identified 195 liver enriched proteins. The HPA identified 69 proteins showing strong liver tissue staining with annotated expression. A total of 272 (37%) proteins were identified in two or more than two databases and therefore selected for further filtration, eliminating approximately 63% of the proteins (Table 1). A complete list of proteins identified by each database is presented in S2 Table.

**Table 1:** Total number of liver-specific proteins identified in gene and protein databases.

Parameters	Liver Specific
Total number of proteins identified	731
(in ≥ two databases)	272
**Number of proteins identified by individual databases**	
One database	459
Two databases	77
Three databases	81
Four databases	64
Five databases	26
Six databases	24

### Liver-specific secreted/shed proteins

Most of the currently known biomarkers for cancer are secreted or shed proteins and it is expected that secreted or shed proteins have the highest chance to reach the circulation [58]. According to our results, majority of the proteins identified in two or more databases were designated as secreted or shed. The number of times each protein is identified in all databases is presented in S3 Table. In total, 208 out of 272 proteins identified as liver-specific were designated as secreted or shed. SignalP (version 4.1) software identified 128 proteins being secreted based on classical secretory mechanism. SecretomeP identified 86 proteins as secretory proteins based on non-classical secretory mechanism. ExoCarta database identified 82 proteins being released via exosomes. 18 proteins were excluded from combined set of Signalp 4.1, SecretomeP 2.0 and Exocarta predicted secretory proteins after scanning via TargetP and TMHMM server. A complete list is given in the S4 Table.

### Expression profile verification

Manual verification of the expression profiles of those secreted or shed proteins identified in two or more than two databases eliminated 170 proteins. Only 5% of the 731 proteins initially identified as highly specific to liver were found to meet the filtering criteria. 38 proteins were filtered out to be liver-specific and secreted or shed therefore represent potential candidate biomarkers.

### Evaluation of the used databases

**Fig 3:**
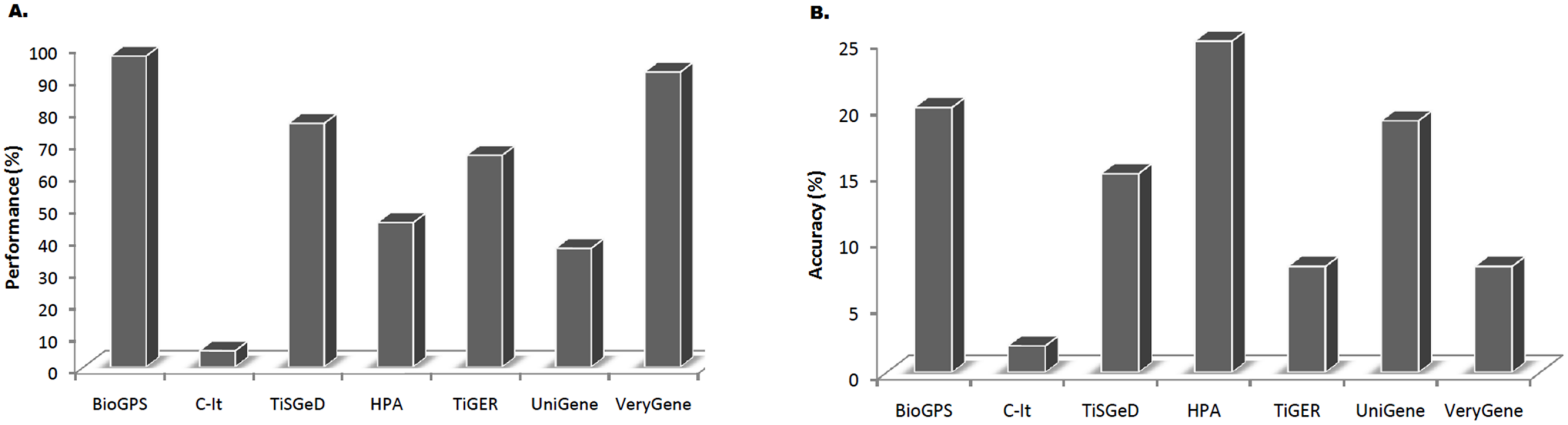
Performance and accuracy evaluation (%) of databases. **3A**. Graphical representation of databases performance has been shown in percentages. BioGPS database revealed 97%, VeryGene database 92%, TiSGeD database 76%, TiGER database 66%, UniGene database 37%, C-It database 5% and the HPA unveiling 45% performance for the identification of liver-specific protein biomarkers. Performance % was calculated by dividing number of proteins identified by each database to total number of proteins that passed the filtering criteria. **3B**. Graphical representation of accuracy of the initial protein identifications with HPA database showing the highest accuracy of 25%, VeryGene database showing 8% accuracy, TiSGeD database showing 15%, TiGER database showing 8%, UniGene database showing 19%, C-It showing 2% and BioGPS database showing 20% accuracy. The accuracy was calculated by dividing number of proteins that had passed the filtering criteria by each database to the total number of proteins each database initially identified.

The performance of the databases was evaluated by determining how many of the 38 proteins that passed the filtering criteria were initially identified by each database (Table 2) (Fig 3A). The BioGPS database identified the greatest number of proteins that passed the filtering criteria (37 out of 38). The VeryGene database identified 35 of the 38 proteins. The TiSGeD database identified 29 of the 38 proteins. The TiGER database had identified 25 of the 38 proteins. The UniGene database identified 14 of the 38 proteins. The C-It database identified 2 of the 38 proteins. The HPA identified 17 of the 38 proteins. The accuracy of the initial protein identifications was evaluated by comparing the proportion of proteins that had passed the filtering criteria to the total number of proteins each database initially identified (Fig 3B). The HPA database showed the highest 25% (17 of 69) accuracy of initial protein identification. The VeryGene database showed 8% accuracy (35 of 465), TiSGeD database showed 15% (29 of 195), TiGER database showed 8% (25 of 309), UniGene database showed 19% (14 of 75), C-It showed 2% (2 of 89) and BioGPS database showed 20% accuracy (37 of 185).

**Table 2:** Liver-specific secreted/shed proteins identified by each database utilized in this study.

Gene	TiSGeD	TiGER	UniGene	C-it	VeryGene	BioGPS	HPA	Reference
ADH6	√	√			√			Secretome of primary human hepatocytes [37]
ANG	√	√			√	√		Previously studied as biomarker [59]
APOA5			√		√	√	√	Secretome of HCC cell line [36]
APOC3	√	√			√	√		Secretome of primary human hepatocytes [37]
APOC4	√	√	√		√	√	√	
APOF	√		√		√	√	√	
ASL	√					√		Secretome of primary human hepatocytes [37]
C4A	√	√				√		Previously studied as biomarker [1]
C8A	√	√	√		√	√	√	Secretome of HCC (Hep3B, serum) [40, 44]
CFHR4	√				√	√	√	Previously studied as biomarker [60]
CYP2A6	√	√	√		√	√	√	Secretome of primary human hepatocytes [37]
CYP2A7	√	√			√	√		
CYP2C18		√			√	√		
CYP2E1	√	√	√		√	√	√	
CYP4A22	√				√	√		
F10	√				√	√		Secretome of HCC cell lines [36, 41]
F9		√	√		√	√	√	
FCN2	√				√	√		
GCKR	√				√	√		
GSTM1	√				√	√		Normal and HCC liver tissue [37]
HAMP	√				√	√		
HP	√	√	√		√	√	√	Previously studied as biomarker [61]
HRG	√	√	√		√	√	√	Previously studied as biomarker [37]
HSD11B1	√				√	√		Secretome of primary human hepatocytes [37]
ITIH4	√	√			√	√	√	Previously studied as biomarker [62]
MBL2			√		√	√	√	Secretome of HCC cell lines (Hep3B, HepG2) [41]
NR0B2	√	√				√		
PLGLB2		√	√		√	√	√	
PON3	√	√			√	√		
RDH16	√	√			√	√	√	Secretome of primary human hepatocytes [37]
SERPINC1	√	√	√		√	√	√	Secretome of HCC serum [43]
SLC25A47			√	√	√	√	√	
SLC27A5	√	√			√	√		
SPP2		√	√	√	√	√	√	
TFR2		√			√	√		Secretome of HCC (Cell line Hep3B) [40, 41]
TMPRSS6		√			√	√		
UPB1	√	√			√	√		Secretome of primary human hepatocytes [37]
VTN	√	√			√	√		Previously studied as biomarker [63, 64]

### Proteins reported in plasma proteome database and liver proteomic datasets

Plasma proteome database further reduced the number of proteins from 38 to 33. Out of these 33 candidate biomarkers, 20 proteins were identified in proteomic datasets enriched with cancer secretome with 6 proteins namely vitronectin (VTN), inter-alpha-trypsin inhibitor heavy chain family, member 4 (ITIH4), haptoglobin (HP), Histidine-rich glycoprotein (HRG), complement component 4A (C4A) and angiogenin (ANG), being previously studied and characterized as circulating HCC biomarkers.

### Functional classification and interactome network analysis

**Fig 4:**
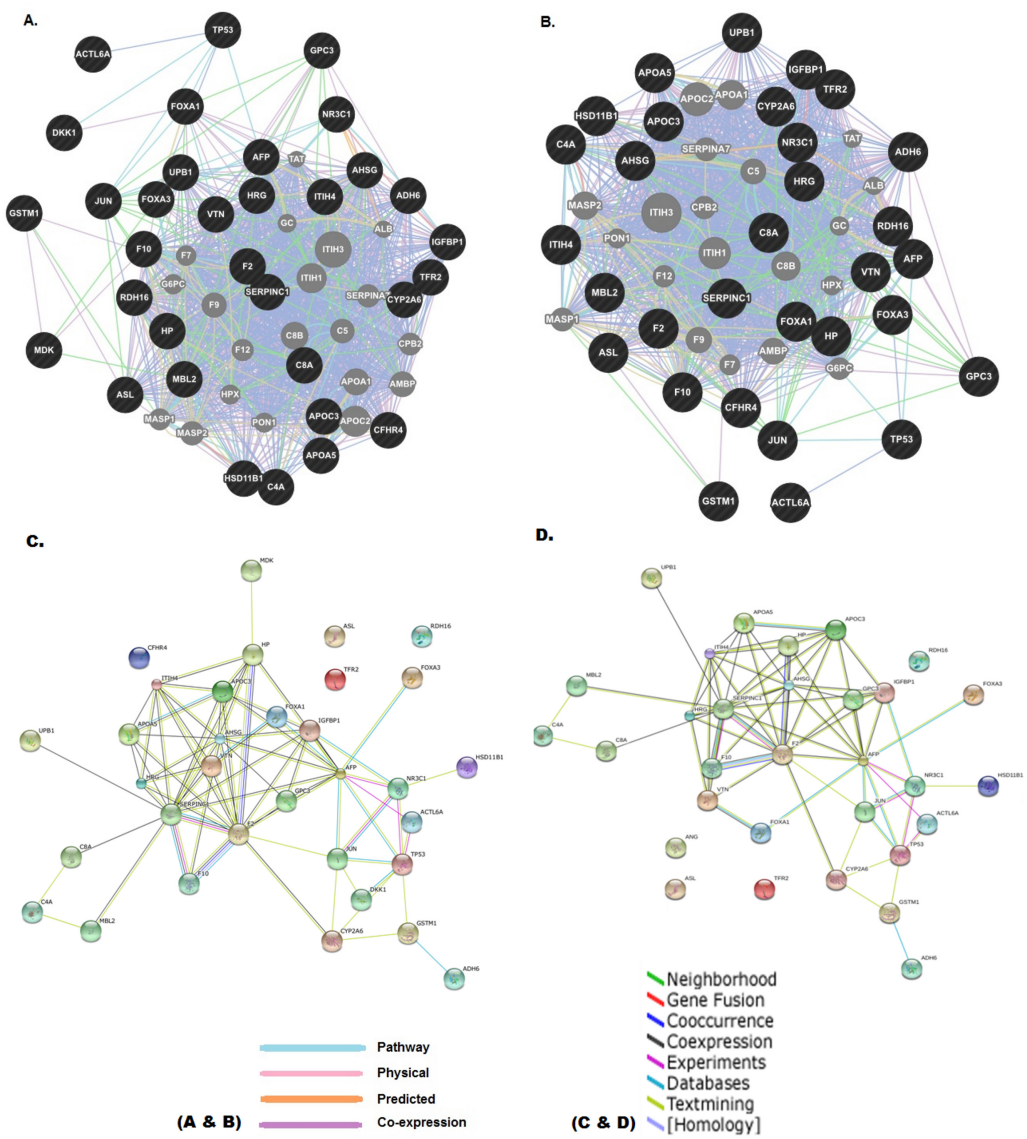
Interactome network analysis (protein-protein). Interactome analysis of candidate proteins with current standard HCC biomarker AFP was retrieved by tools: GeneMANIA (A & B), STRING (C & D). Interacting partners of AFP (TP53, FOXA1, FOXA3, GPC3, IGFBP1, NR3C1, F2, AHSG, ACTL6A and JUN) along with DKK1 and MDK are also main elements of the interactome. The size of the gray nodes in Fig 4A and 4B represents the degree of association with the input genes (i.e., smaller size represents less association).

Interactome analysis with current standard HCC biomarker AFP, its interacting partners (TP53, FOXA1, FOXA3, GPC3, IGFBP1, NR3C1, F2, AHSG, ACTL6A and JUN) along with DKK1 and MDK filtered 11 candidates ADH6, APOA5, APOC3, C8A, CYP2A6, F10, GSTM1, HSD11B1, MBL2, SERPINC1 and UPB1 as potential circulating biomarkers (Fig 4). Studies have shown the potential of AFP not only as a diagnostic marker but also as a growth factor in promoting pathological progression of HCC through P13K/AKT signaling pathway [65, 66]. Li et al. also reported the interaction of AFP with caspase-3 in the cytoplasm which ultimately blocks the apoptotic signaling pathway by impeding onward transmission of signaling from caspase-8 [67]. Rationale behind the use of DKK1 and MDK is that they are recent reliable serum biomarkers and are expected to be used clinically to facilitate screening for and diagnosing HCC at an earlier stage. The 11 candidate proteins appeared at the fulcrum of the functional network, suggesting possible association with HCC progression. Cancer atlas results of HPA were also integrated in further filtration process.

**Fig 5:**
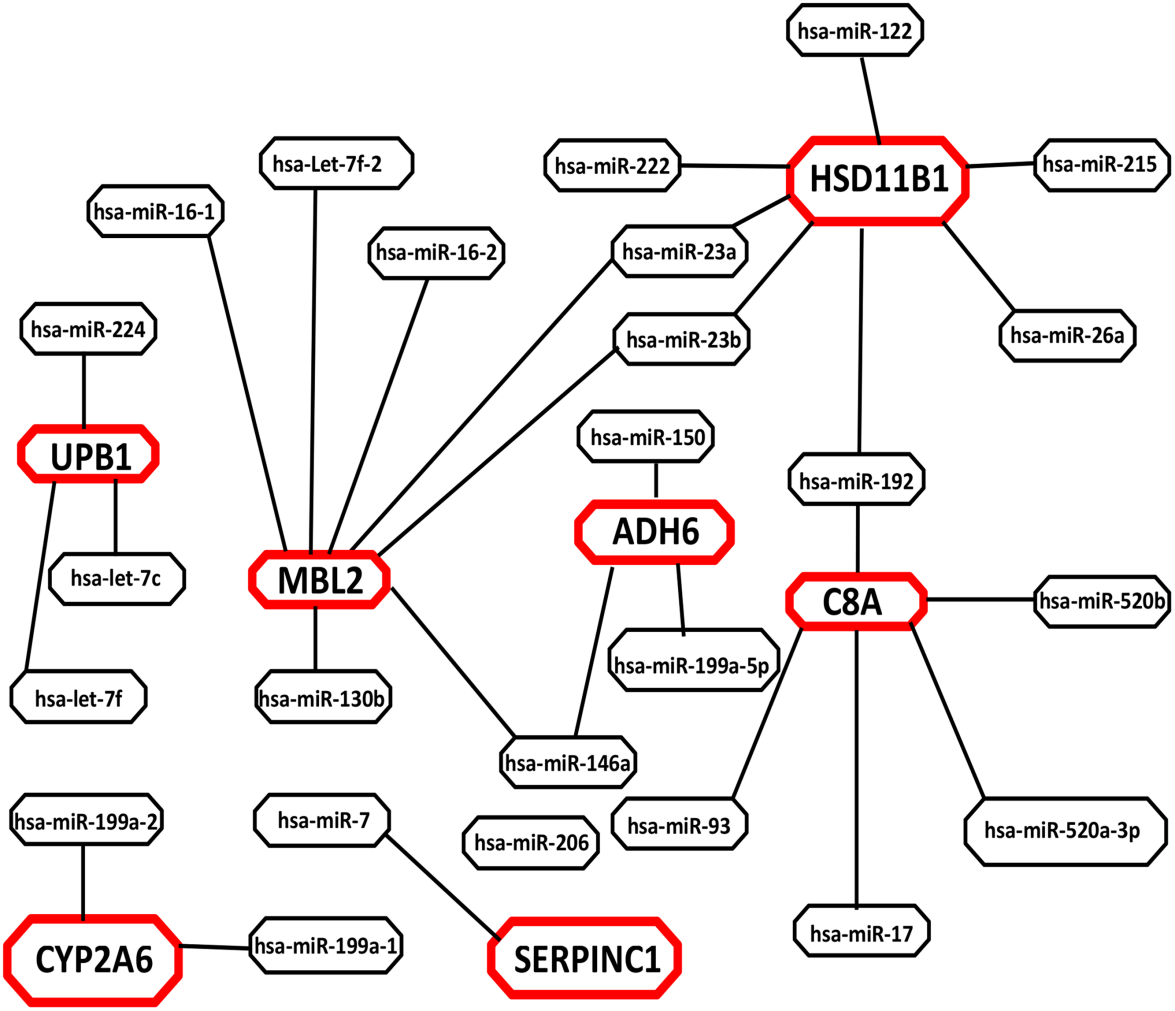
Interactome network analysis (miRNA-gene). Interactome network analysis of HCC-specific circulating miRNAs and genes encoding candidate protein biomarkers (retrieved using miRWalk, miRTarBase, TargetScan and microRNA.org) were visualized using Cytoscape software. The red colored circles represent seven final prioritized candidate marker proteins in our study.

Candidate proteins were further enriched on the basis of their encoding genes as validated targets of HCC-specific circulating and liver deregulated miRNAs, as differential expression of miRNAs can affect the expression of their target genes, leading to changes in the levels of proteins they encode [68], prioritizing seven proteins as candidate biomarkers (S6 Table). Interactome analysis of seven putative candidates with HCC-specific circulating miRNAs (hsa-miR-30c, hsa-miR-520b, hsa-miR-150 [69], hsa-miR-130b [70], hsa-miR-1 [71], hsa-miR-192, hsa-miR-26a [72, 73], hsa-miR-7, hsa-let-7f [72], has-miR-224 [74], hsa-miR-199a-5p [75], hsa-miR-23a, hsa-miR-23b, hsa-miR-146a [76], hsa-miR-206 [77], hsa-miR-215, hsa-miR-93, hsa-miR-17, hsa-miR-520a-3p [78]) has been shown in Fig 5 (Table 3).

**Table 3:** Seven statistically significant putative HCC specific biomarkers prioritized through integrated *in-silico* approach.

Gene	Biological functions	HCC-specific deregulated miRNAs (Liver) (Circulating)	
ADH6	Metabolism of xenobiotics by cytochrome P450, drug metabolism, glycolysis/gluconeogenesis, tyrosine metabolism, metabolic pathways, retinol metabolism and fatty acid metabolism.	hsa-miR-182, hsa-miR-185, hsa-miR-203, hsa-miR-199a-5p, hsa-miR-199b-5p, hsa-miR-146a, hsa-miR-211, hsa-miR-150	hsa-miR-199a-5p, hsa-miR-146a, hsa-miR-150
UPB1	Beta alanine metabolism, metabolic pathway, pantotheanate and CoA biosynthesis, drug metabolism-other enzymes and pyrimidine metabolism.	hsa-miR-216a, hsa-miR-181c, hsa-miR-181a, hsa-miR-181b, hsa-miR-134, hsa-let-7e, hsa-let-7b, hsa-let-7a, hsa-let-7c, hsa-let-7f, hsa-let-7g, hsa-let-7d, hsa-miR-224	hsa-let-7f, hsa-let-7c, hsa-miR-224
C8A	Complement and coagulation cascades, prion disease, systemic lupus erythematosus and amoebiasis.	hsa-miR-212, hsa-miR-132, hsa-miR-93, hsa-miR-106a, hsa-miR-106b, hsa-miR-17, hsa-miR-20a, hsa-miR-302b, hsa-miR-26a, hsa-miR-26b, hsa-miR-145, hsa-miR-148a, hsa-miR-148b, hsa-miR-152, hsa-miR-186, hsa-miR-129-5p	hsa-miR-93, hsa-miR-17, hsa-miR-520a-3p, hsa-miR-520b, hsa-miR-26a,
HSD11B1	Steroid hormone biosynthesis, metabolic pathways, and in aldosterone-regulated sodium reabsorption.	hsa-miR-181c, hsa-miR-181a, hsa-miR-181b, hsa-miR-374a, hsa-miR-374b, hsa-miR-192, hsa-miR-215, hsa-miR-23a, hsa-miR-23b, hsa-miR-132, hsa-miR-212, hsa-miR-26a, hsa-miR-26b, hsa-mir-122, hsa-mir-125a, hsa-mir-125b-1, hsa-mir-125b-2, hsa-mir-145, hsa-mir-222	hsa-miR-192, hsa-miR-215, hsa-miR-23a, hsa-miR-23b, hsa-miR-26a, hsa-mir-122, hsa-mir-222
MBL2	Complement and coagulation cascades, acute-phase response, classical pathway, negative regulation of viral process, opsonization, positive regulation of phagocytosis.	hsa-miR-320c, hsa-miR-374b, hsa-miR-374a, hsa-miR-186, hsa-miR-200b, hsa-miR-301a, hsa-miR-301b, hsa-miR-137, hsa-miR-23a, hsa-miR-23b, hsa-miR-206, hsa-miR-216b, hsa-miR-30a, hsa-miR-30e, hsa-miR-30c, hsa-miR-146a, hsa-miR-190b, hsa-miR-190, hsa-miR-130a, hsa-miR-130b, hsa-miR-148a, hsa-miR-148b, hsa-miR-152, hsa-miR-145, hsa-miR-196a, hsa-miR-216a, hsa-miR-1, hsa-let-7a-2, hsa-let-7a-3, hsa-let-7a-1, hsa-let-7b, hsa-let-7c, hsa-let-7d, hsa-let-7e, hsa-let-7f-2, hsa-let-7g, hsa-mir-1-2, hsa-mir-1-1, hsa-mir-10a, hsa-mir-125b-1, hsa-mir-125b-2, hsa-mir-15a, hsa-mir-16-1, hsa-mir-16-2, hsa-mir-7-1, hsa-mir-7-2, hsa-mir-7-3, hsa-mir-99a	hsa-miR-23a, hsa-miR-23b, hsa-miR-30c, hsa-miR-146a, hsa-miR-130b, hsa-miR-1, hsa-miR-206, hsa-let-7f-2, has-let-7c, hsa-mir-16-1, hsa-mir-16-2
SERPINC1	Complement and coagulation cascades, neuroactive ligand receptor interaction and regulation of actin cytoskeleton.	hsa-miR-186, hsa-miR-19a, hsa-miR-19b, hsa-miR-143, hsa-miR-7	hsa-miR-7
CYP2A6	Drug metabolic process, epoxygenase P450 pathway, exogenous drug catabolic process, oxidation-reduction process, small molecule metabolic process, steroid metabolic process, xenobiotic metabolic process.	hsa-mir-101-1, hsa-mir-101-2, hsa-mir-126, hsa-mir-199a-1, hsa-mir-199a-2, hsa-mir-199b, hsa-mir-34a	hsa-mir-199a-1, hsa-mir-199a-2

### SurvExpress results of candidate biomarkers

**Fig 6:**
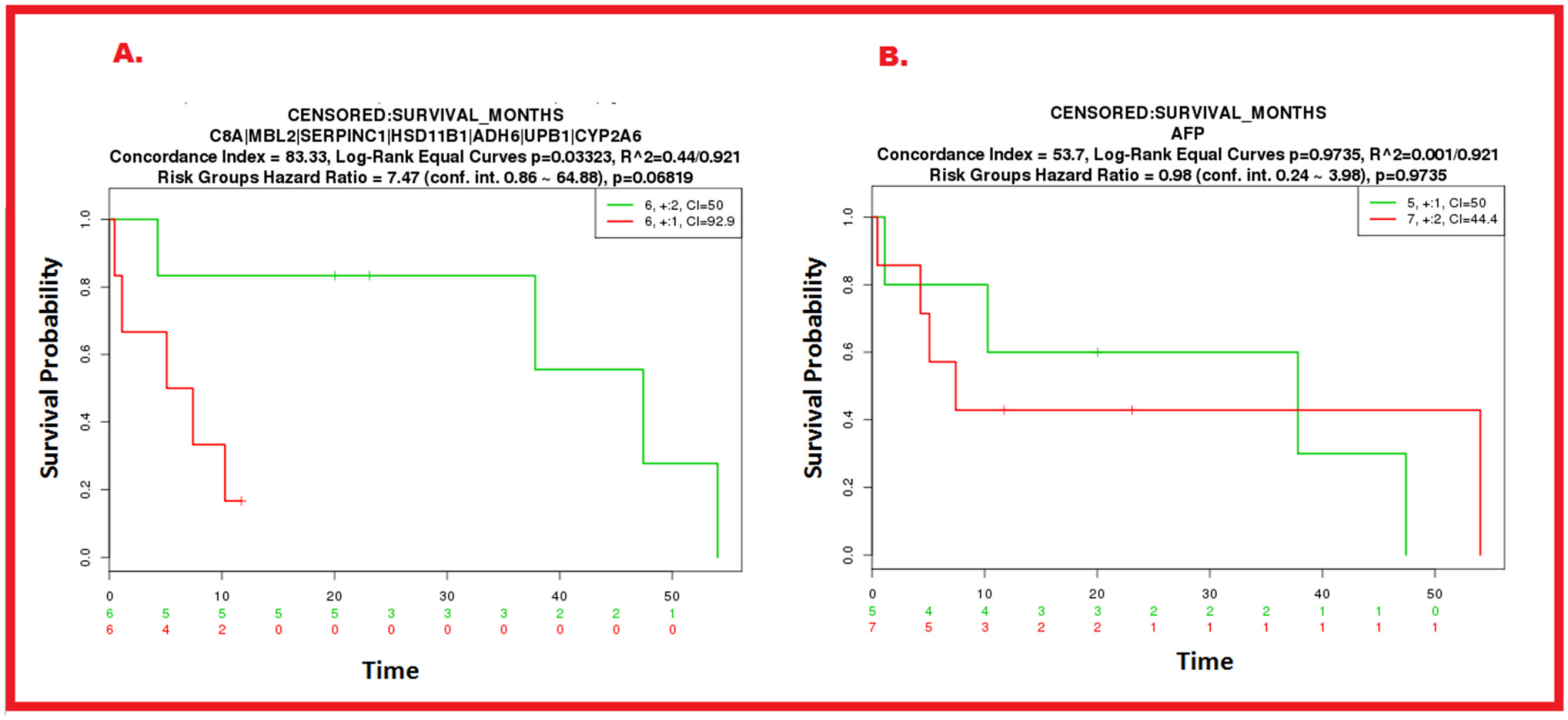
Comparison of Kaplan-Meier curves of the current standard HCC biomarker (AFP) and candidate seven circulating biomarkers (C8A, MBL2, SERPINC1, HSD11B1, ADH6, UPB1, CYP2A6). SurvExpress analysis showed the results from liver hepatocellular carcinoma dataset using TCGA RNASeq platform of SurvExpress. **A** shows the Kaplan-Meier curve for risk groups, concordance index, and P-value of the log-rank testing equality of survival curves for AFP. **B** shows the Kaplan-Meier curve for risk groups, concordance index, and P-value of the log-rank testing equality of survival curves for C8A, MBL2, SERPINC1, HSD11B1, ADH6, UPB1 and CYP2A6.

SurvExpress results revealed CI of AFP to be 53.7 whereas for our proposed seven biomarkers (C8A, MBL2, SERPINC1, HSD11B1, ADH6, UPB1, CYP2A6), SurvExpress showed CI value of 83.33. CI values close to 0.5 are putatively ‘random’ whereas higher values are associated to better prediction. Expression of AFP in the tumor compartment was not statistically significant with longer RFS (log rank P = 0.2) whereas the expressions of C8A, MBL2, SERPINC1, HSD11B1, ADH6, UPB1 and CYP2A6 were statistically significantly with longer RFS (log rank P = 0.03) as shown in Fig 6.

**Fig 7:**
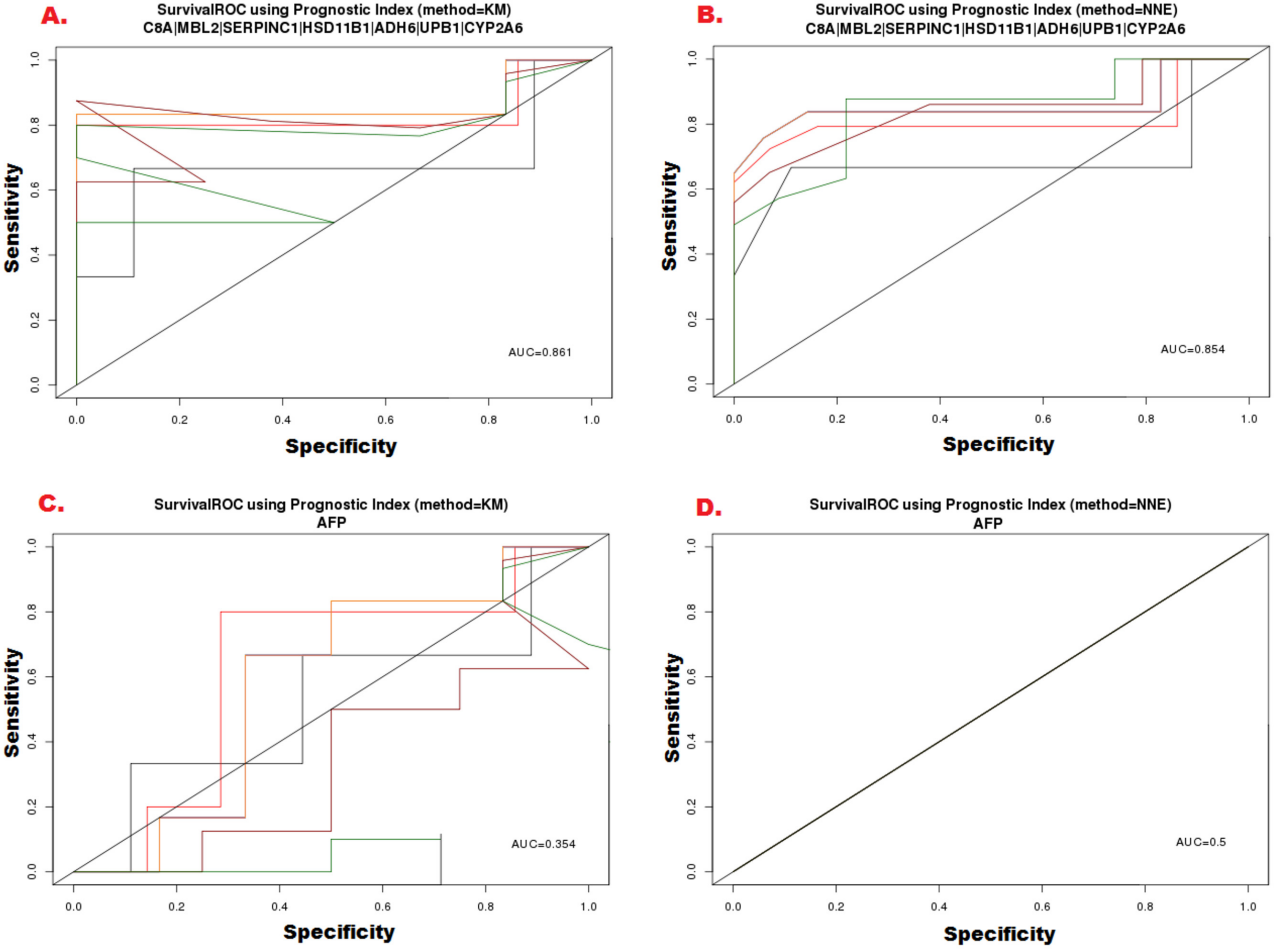
Receiver operating characteristic (ROC) analysis of sensitivity and specificity by proposed seven candidate biomarkers and AFP in predicting disease-free survival (DFS). The score performance was assessed by calculating the area under the ROC (AUROC) which was 0.861 (KM method) and 0.854 (NNE method), respectively for proposed candidate biomarkers while for AFP; AUROC was 0.354 (KM method) and 0.5 (NNE method) respectively.

**Fig 8:**
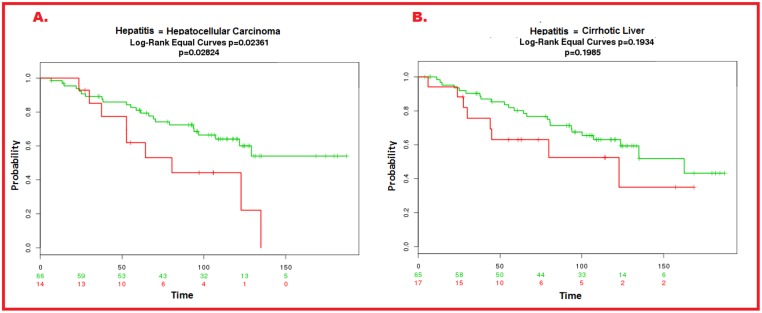
Relapse-free survival and ROC curve analysis. Proposed candidate biomarkers better predicted relapse-free survival (p = 0.01191) **(A)** as compared to AFP (P = 0.1987) **(B)**. With respect to the discriminating ability of proposed biomarkers, long rank equal curve showed statistically significant p-value (<0.05) for HCC p = 0.02361 while for cirrhotic liver p-value was 0.1985 (which not significant) (Fig 9A and 9B).

In predicting disease and relapse-free survival, the proposed candidate biomarkers performed better comparing to AFP as shown by the Kaplan-Meier method and the ROC curves analysis (Figs 7 and 8). The ROC curves documented a significant statistical correlation of the proposed candidate biomarkers with MBL2, C8A, SERPINC1, HSD11B1, ADH6, UPB1 and CYP2A6 levels predicting HCC-free survival considerably well {area under ROC = 0.861 (KM), and area under ROC = 0.854 (NNE)}, while no significance was found for AFP and HCC-free survival rate (area under ROC = 0.354 (KM), and area under ROC = 0.5 (NNE).

**Fig 9:**
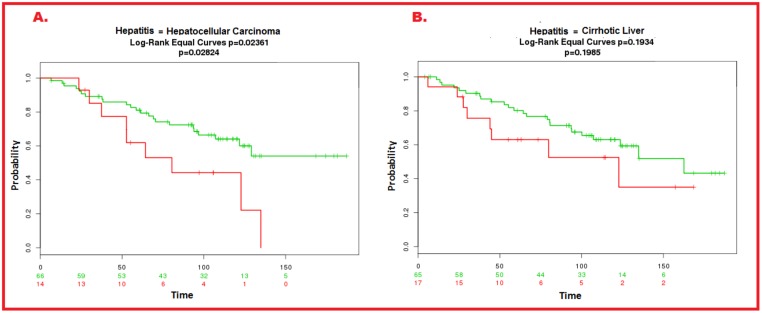
ROC curve analysis of proposed candidate biomarkers in HCC and cirrhotic datasets. With respect to HCC, the candidate biomarkers showed statistically significant relation (p = 0.02824) (A) while for cirrhosis there was no significant correlation (p = 0.1985) (B).

For further confirmation we also analyzed the existing experimental data on circulating cirrhotic markers and performed a meta-analysis. The proposed biomarkers were investigated in the studies related to circulating biomarkers for cirrhotic patients in order to assess whether these proteins were present in their list or not. None of the predicted proteins appeared in their study, suggesting that our proposed biomarkers are not specific for cirrhosis, further confirming the reliability of our proposed pipeline.

## Discussion

HCC ranks third in overall cancer related mortality worldwide. The discovery of novel circulating biomarkers is expected to facilitate screening and diagnosis of HCC at an earlier stage which will help in limiting HCC related morbidity and mortality [7]. The importance of highly sensitive and more specific clinical biomarkers for HCC has been well established therefore we endeavored to design a performance-based study to identify and evaluate predictive and prognostic biomarkers. The pipeline integrates various bioinformatics tools, databases and literature to comprehensively analyze vast proteomics expression data in order to find highly sensitive and specific protein biomarkers. The study identifies seven important proteins including C8A (complement component 8, alpha polypeptide), MBL2 (mannose binding lectin 2), SERPINC1 (Antithrombin III), HSD11B1 (11β-hydroxysteroid dehydrogenase type 1), ADH6 (Alcohol dehydrogenase 6), UPB1 (Beta-ureidopropionase) and CYP2A6 (Cytochrome P450, family 2, subfamily A, polypeptide 6). These predicted proteins are novel, highly specific and sensitive, which may serve as more efficient clinical biomarkers in case of HCC. Additionally, these proteins also satisfy the following criteria for example, liver-specificity, secretory nature, verified expression in liver and blood, presence in liver secretome, direct or indirect interaction with AFP, MDK, DKK1 and encoded by genes which are validated targets of HCC-specific circulating and liver deregulated miRNAs. All of the prioritized proteins were critically evaluated based on literature data and experimental evidences in order to analyze their biological role and significance as probable clinical biomarker.

Alcohol dehydrogenase 6 (ADH6) is among one of the prioritized biomarker which encodes class V alcohol dehydrogenase (ADH). Several studies showed elevated level of ADH in sera of liver cancer patients [79, 80]. Moreover, various cancer studies (secretome analysis) have reported differential expression of ADH6 in HCC-specific cell lines, sera and liver tissues indicating its specificity and sensitivity in detecting HCC. Unlikely, ADH6 also showed up to five fold decreased expression in the HCC secretome analysis as compared to normal [37] (Table 2) (S7 Table). Furthermore, a PPI network analysis revealed direct interaction of ADH6 with GSTM1 (Fig 4). GSTM1 is one of well-studied metabolic gene and is an interacting partner of TP53 and CYP2A6. GSTM1 belongs to GSTs family which plays a regulatory role in MAP kinase pathway (cellular survival and death signaling) and are involved in various cancers [81]. This is also evident from the fact that ∼50% of HCC patients are TP53 positive [82] and detection of serum TP53 along with AFP increased the frequency of HCC prediction from 79.5% (AFP only) to 86.3% (AFP and p53). As ADH6 is an interacting partner of GSTM1 and is more specific to liver, thus it is highly probable that ADH6 can be a good biomarker candidate. Additionally, ADH6 is also targeted by three circulating and eight liver deregulated miRNAs (Fig 5) (Table 3) suggesting its possible involvement in HCC pathogenesis.

Another protein identified as potential biomarker is mannose binding lectin 2 (MBL2). It is an acute phase reactant that is secreted from liver and is critical in host defenses against a spectrum of viral, bacterial, fungal and parasitic pathogens. MBL deficiency has been associated with a range of auto-immune and infectious diseases, including HIV-1 and hepatitis B viral infections [83, 84]. It was also revealed through literature that MBL2 has strong secretory nature [41] and strong presence in liver cancer tissue and cell line (HepG2). Serum levels of MBL2 have been reported to be significantly higher in pancreatic cancer patients [85] suggesting possible involvement in cancer progression. PPI network analysis further strengthened its potential as a candidate biomarker because MBL2 showed direct interaction with MDK, C4A and SERPINC1 (Fig 4). Interestingly, C4A and MDK are previously characterized circulating biomarkers for HCC [1] whereas SERPINC1 (also prioritized as a potential biomarker in our study) is an interacting partner of AFP, C8A, F2, UPB1, F10, AHSG, APOA5, APOC3 and IGFBP1 (involved in cancer progression) (Fig 4). MBL2 have also been shown to be involved in complement & coagulation cascades and phagosomes. As a matter of fact complement and coagulation cascade has been reported to be the most perturbed pathway in various cancers [86, 87] strengthening the notion that MBL2 may serve as a good predictive and prognostic clinical biomarker. Current study also revealed that gene encoding MBL2 protein is a target of eleven circulating and forty-eight liver deregulated miRNAs (Fig 5) (Table 3). These findings strongly suggest that MBL2 should be further validated and characterized as a biomarker for HCC.

Furthermore, antithrombin III (SERPINC1) is a serine proteinase inhibitor which controls the process of coagulation. SERPINC1 was found to be differentially expressed between serum of HCC patients and healthy subjects [43]. Likewise, complement component 8, alpha (C8A), one of the end terminals of the complement system in the membrane attack complex (MAC), is also a potential biomarker. C8A has been reported to be present in the secretome of HCC cell line HEP3B [40]. Another protein, 11β-hydroxysteroid dehydrogenase type 1 (HSD11B1), a primary reductase, is an NADPH-dependent microsomal enzyme, highly expressed in liver and is also a biomarker candidate prioritized in the current study. Its presence in primary human hepatocytes secretome was confirmed in a study conducted by Wang et al. revealing its potential as a secretory protein [37]. CYP2A6 was also found to be strongly associated with HCC and liver secretome as validated by various experimental evidences present in literature. Beta-ureidopropionase (UPB1) catalyzes the last step in the pyrimidine degradation pathway. Cancer tissue and cell line atlas of HPA also showed strong antibody staining of these proteins in liver cancer and in HCC cell line HepG2. Interactome analysis revealed direct or indirect interaction of these proteins with current standard HCC biomarker AFP as well as with other important proteins which are either previously characterized as HCC biomarkers or are involved in cancer pathogenesis (Fig 4).

In order to analyze the interactions between the target genes encoding candidate proteins and HCC-specific circulating miRNAs, a miRNA and corresponding gene network was built based on the hypothesis that differential expression of miRNAs can affect the expression of their target genes, leading to changes in the levels of proteins they encode [68]. miRNA-gene interactome (Fig 5) (Table 3) revealed that C8A and HSD11B1 are the common target genes of hsa-miR-26a where as HSD11B1 was also found to be the target gene of hsa-miR-122 and hsa-miR-192. Zhou et al. showed that a panel of miRNAs (hsa-miR-122, hsa-miR-192, hsa-miR-21, hsa-miR-223, hsa-miR-26a and hsa-miR-27a) has considerable clinical importance in diagnosing early-stage HCC [73]. SERPINC1, MBL2 and UPB1 were found to be common target genes of hsa-let-7 family with MLBL2 and UPB1, common target genes of hsa-let-7f and hsa-let-7c; SERPINC1 as a target gene for has-let-7. hsa-miR-7f and has-let-7c are shown to be highly expressed in the serum of HBV-positive HCC and have been studied as biomarker for HBV induced HCC [77, 88]. MBL2 was also found to be the target gene of has-miR-130b. Studies have shown has-miR-130b as a circulating miRNA originating from tumor. Its level is significantly up-regulated in HCC tissues, cell lines and serum samples. Post-surgery analysis of HCC Serum level of hsa-miR-130b also showed down-regulation [70, 88]. HSD11B1 and MBL2 were observed as common target genes of hsa-miR-23a and hsa-miR-23b. MBL2 was also found to be the target gene of hsa-miR-146a. Serum level of hsa-miR-146a was significantly down-regulated in HCC patients [76]. Interactome analysis also revealed MBL2 as a target gene of has-miR-16. Qu et al. found serum level of has-miR-16 having highest sensitivity for HCC followed by hsa-miR-199a, AFP, DCP and AFP-L3 [75]. ADH6 and CYP2A6 were observed as common target genes of hsa-miR-199a. has-miR-199a was shown to be significantly reduced in HCC serum samples [75].

This data suggests that these proteins are highly specific and sensitive to liver tissue and can be detected easily in the serum of the patients. Due to their differential expression in normal and diseased state, they can be used as clinical biomarkers. It is suggested that instead of using a single biomarker, combination of multiple biomarkers may increase diagnostic sensitivity and specificity [89]. Our proposed prioritization pipeline also unveiled 6 previously characterized circulating HCC biomarkers including VTN, ITIH4, HP, HRG, C4A and ANG, further increasing the reliability of our strategy. The protein signatures should be investigated in cohort studies with a large numbers of patients in order to verify the potential use of these above mentioned proteins as clinical biomarkers.

## Conclusions

High-throughput quantitative proteomics technology and a combination of computational methods have provided a technological advancement for identifying tumor markers. Our prioritization strategy has identified seven potential putative circulating protein biomarkers for HCC which are encoded by the target genes of HCC-specific liver deregulated and circulating miRNAs. Given the heterogeneity and complexity of etiology and clinical behaviors of HCC, it would be very difficult to find single biomarker that is both specific and sensitive enough. Combination of pathological features and biomarkers with high sensitivity and specificity seems to be more practical for early diagnosis and prognostication of HCC. Further experimental studies are necessary to validate our proposed novel biomarkers in human subjects in order to elucidate the role of these proteins as circulating biomarkers and their role in HCC pathogenesis and progression. We have demonstrated an unbiased bioinformatics and proteomics filtering strategy to objectively identify a set of proteins which are attractive candidates for biomarker testing. Current study has demonstrated that an integrative secretome, interactome and miRNAs target filtration strategy can be used as an effective screening approach to effectively extract valuable new insight from the huge number of existing datasets. Our pipeline is straight forward, user-friendly and can be extended explicitly to other cancer biomarker studies.

## Supporting Information

S1 Table List of databases and tools used to prioritize circulating biomarkers for HCC. (DOCX)

S2 Table List of liver-specific proteins identified by seven renowned databases. Results of each database have been represented in separate columns. Proteins declared to be liver-specific in more than 2 databases were selected for further analysis.
(XLSX)

S3 Table List of proteins identified in more than two databases used in this study. Liver-specific proteins were identified through several databases (S1 Table). Proteins identified in more than 2 databases are listed in this file.
(XLSX)

S4 Table List of Secreted/shed proteins. To prioritize secretory protein biomarkers specific for HCC, all prioritized protein were checked for the presence of secretory signal using a pipeline of five tools; SignalP 4.1, SecretomeP 2.0, ExoCarta, TargetP 1.1 and TMHMM v. 2.0.
(XLSX)

S5 Table List of liver-specific secreted proteins with verified expression profiles. Secreted or shed liver-specific proteins (S3 Table) with verified expression profiles in liver and blood are listed along with their full names.
(XLSX)

S6 Table Liver-specific proteins encoded by target genes of HCC-specific circulating and liver deregulated miRNAs. (DOCX)

S7 Table List of liver-specific proteins which have not been previously studied/ characterized as circulating biomarker for HCC. (DOCX)
