# Supplementary material for: Identification of Circulating Biomarker Candidates for Hepatocellular Carcinoma (HCC): An Integrated Prioritization Approach
Source: PLoS One. 2015 Sep 28;10(9):e0138913. doi: 10.1371/journal.pone.0138913 (PMC4586137; doi:10.1371/journal.pone.0138913)
Supplement: S1 Table — (DOCX) [file pone.0138913.s001.docx]

| Databases/Tools/Softwares | Accessibility |
| --- | --- |
| TiSGeD | <http://bioinf.xmu.edu.cn:8080/databases/TiSGeD/index.html> |
| VeryGene | <http://www.verygene.com/> |
| UniGene | <http://www.ncbi.nlm.nih.gov/unigene> |
| TiGER | <http://bioinfo.wilmer.jhu.edu/tiger/> |
| C-It | <http://c-it.mpi-bn.mpg.de/> |
| BioGPS | <http://biogps.org/#goto=welcome> |
| The Human Protein Atlas | <http://www.proteinatlas.org/> |
| UniProtKB | <http://www.uniprot.org/> |
| SignalP 4.1 | <http://www.cbs.dtu.dk/services/SignalP/> |
| SecretomeP 2.0 | <http://www.cbs.dtu.dk/services/SecretomeP/> |
| ExoCarta | <http://www.exocarta.org/> |
| TargetP 1.1 | <http://www.cbs.dtu.dk/services/TargetP/> |
| TMHMM v. 2.0 | <http://www.cbs.dtu.dk/services/TMHMM/> |
| Plasma Proteome Database | <http://www.plasmaproteomedatabase.org/> |
| GeneMANIA | <http://www.genemania.org/> |
| STRING | <http://string-db.org/> |
| FpClass | <http://dcv.uhnres.utoronto.ca/FPCLASS/ppis/> |
| MiRWalk | <http://www.umm.uni-heidelberg.de/apps/zmf/mirwalk/> |
| miRTarBase | <http://mirtarbase.mbc.nctu.edu.tw/> |
| TargetScan | <http://www.targetscan.org/> |
| MicroRNA.org | <http://www.microrna.org/microrna/home.do> |
| Cytoscape | <http://www.cytoscape.org/> |
| SurvExpress | [http://bioinformatica.mty.itesm.mx:8080/Biomatec/Surviva X.jsp](http://bioinformatica.mty.itesm.mx:8080/Biomatec/Surviva%20X.jsp) |
