# Supplementary material for: Identification of Circulating Biomarker Candidates for Hepatocellular Carcinoma (HCC): An Integrated Prioritization Approach
Source: PLoS One. 2015 Sep 28;10(9):e0138913. doi: 10.1371/journal.pone.0138913 (PMC4586137; doi:10.1371/journal.pone.0138913)
Supplement: S6 Table — (DOCX) [file pone.0138913.s006.docx]

| **Gene** | **HCC-specific deregulated miRNAs** | |
| --- | --- | --- |
|  | **Liver** | **Circulating** |
| ADH6 | hsa-miR-182  hsa-miR-185  hsa-miR-203  hsa-miR-199a-5p  hsa-miR-199b-5p  hsa-miR-146a  hsa-miR-211  hsa-miR-150 | hsa-miR-199a-5p  hsa-miR-146a  hsa-miR-150 |
| APOA5 | hsa-miR-338-3p  hsa-miR-22  hsa-miR-24  hsa-miR-214 |  |
| APOC3 | hsa-miR-200b |  |
| UPB1 | hsa-miR-216a  hsa-miR-181c  hsa-miR-181a  hsa-miR-181b  hsa-miR-134  hsa-let-7e  hsa-let-7b  hsa-let-7a  hsa-let-7c  hsa-let-7f  hsa-let-7g  hsa-let-7d  hsa-miR-224 | hsa-let-7f  hsa-let-7c  hsa-miR-224 |
| C8A | hsa-miR-212  hsa-miR-132  hsa-miR-93  hsa-miR-106a  hsa-miR-106b  hsa-miR-17  hsa-miR-20a  hsa-miR-302b  hsa-miR-26a  hsa-miR-26b  hsa-miR-145  hsa-miR-148a  hsa-miR-148b  hsa-miR-152  hsa-miR-186  hsa-miR-129-5p | hsa-miR-93  hsa-miR-17  hsa-miR-520a-3p  hsa-miR-520b  hsa-miR-26a |
| HSD11B1 | hsa-miR-181c  hsa-miR-181a  hsa-miR-181b  hsa-miR-374a  hsa-miR-374b  hsa-miR-192  hsa-miR-215  hsa-miR-23a  hsa-miR-23b  hsa-miR-132  hsa-miR-212  hsa-miR-26a  hsa-miR-26b  hsa-mir-122  hsa-mir-125a  hsa-mir-125b-1  hsa-mir-125b-2  hsa-mir-145  hsa-mir-222 | hsa-miR-192  hsa-miR-215  hsa-miR-23a  hsa-miR-23b  hsa-miR-26a  hsa-mir-122  hsa-mir-222 |
| MBL2 | hsa-miR-320c  hsa-miR-374b  hsa-miR-374a  hsa-miR-186  hsa-miR-200b  hsa-miR-301a  hsa-miR-301b  hsa-miR-137  hsa-miR-23a  hsa-miR-23b  hsa-miR-206  hsa-miR-216b  hsa-miR-30a  hsa-miR-30e  hsa-miR-30c  hsa-miR-146a  hsa-miR-190b  hsa-miR-190  hsa-miR-130a  hsa-miR-130b  hsa-miR-148a  hsa-miR-148b  hsa-miR-152  hsa-miR-145  hsa-miR-196a  hsa-miR-216a  hsa-miR-1  hsa-let-7a-2  hsa-let-7a-3  hsa-let-7a-1  hsa-let-7b  hsa-let-7c  hsa-let-7d  hsa-let-7e  hsa-let-7f-2  hsa-let-7g  hsa-mir-1-2  hsa-mir-1-1  hsa-mir-10a  hsa-mir-125b-1  hsa-mir-125b-2  hsa-mir-15a  hsa-mir-16-1  hsa-mir-16-2  hsa-mir-7-1  hsa-mir-7-2  hsa-mir-7-3  hsa-mir-99a | hsa-miR-23a  hsa-miR-23b  hsa-miR-30c  hsa-miR-146a  hsa-miR-130b  hsa-miR-1  hsa-miR-206  hsa-let-7f-2  has-let-7c  hsa-mir-16-1  hsa-mir-16-2 |
| SERPINC1 | hsa-miR-186  hsa-miR-19a  hsa-miR-19b  hsa-miR-143  hsa-miR-7 | hsa-miR-7 |
| CYP2A6 | hsa-mir-101-1  hsa-mir-101-2  hsa-mir-126  hsa-mir-199a-1  hsa-mir-199a-2  hsa-mir-199b  hsa-mir-34a | hsa-mir-199a-1  hsa-mir-199a-2 |
