# Supplementary material for: Identification of Circulating Biomarker Candidates for Hepatocellular Carcinoma (HCC): An Integrated Prioritization Approach
Source: PLoS One. 2015 Sep 28;10(9):e0138913. doi: 10.1371/journal.pone.0138913 (PMC4586137; doi:10.1371/journal.pone.0138913)
Supplement: S7 Table — (DOCX) [file pone.0138913.s007.docx]

| **Gene** | **Protein name** | **Secretome of HCC**  **(Cell lines, liver tissue, serum)** | **Secretome of primary human hepatocytes** |
| --- | --- | --- | --- |
| ADH6 | Alcohol dehydrogenase 6 |  | √ [[36](#_ENREF_36)] |
| APOA5 | Apolipoprotein A-V | Cell line [[35](#_ENREF_35)] |  |
| APOC3 | Apolipoprotein C-III |  | √ [[36](#_ENREF_36)] |
| ASL | Argininosuccinate lyase |  | √ [[36](#_ENREF_36)] |
| C8A | Complement component 8A | Hep3B [[39](#_ENREF_39)]  Serum [[43](#_ENREF_43)] |  |
| CYP2A6 | Cytochrome P450 2A6 |  | √ [[36](#_ENREF_36)] |
| F10 | Coagulation factor X | Hep3B [[40](#_ENREF_40)]  Cell line [[35](#_ENREF_35)] |  |
| GSTM1 | Glutathione S-transferase Mu 1 | Normal and HCC liver tissue [[36](#_ENREF_36)] |  |
| HSD11B1 | Hydroxysteroid (11-beta) dehydrogenase 1 |  | √ [[36](#_ENREF_36)] |
| MBL2 | Mannose-binding lectin (protein C) 2 | Hep3B, HepG2 [[40](#_ENREF_40)] |  |
| RDH16 | Retinol dehydrogenase 16 |  | √ [[36](#_ENREF_36)] |
| SERPINC1 | Serpin peptidase inhibitor, clade C (antithrombin), member 1 | Serum [[42](#_ENREF_42)] |  |
| TFR2 | Transferrin receptor 2 | Hep3B [[40](#_ENREF_40)] [[39](#_ENREF_39)] |  |
| UPB1 | Ureidopropionase, beta |  | √ [[36](#_ENREF_36)] |
